# Low-cost UAV detection via WiFi traffic analysis and machine learning

**DOI:** 10.1038/s41598-023-47453-6

**Published:** 2023-11-28

**Authors:** Longtao Bi, Zi-Xin Xu, Ling Yang

**Affiliations:** College of Electronic Engineering, Chengdu University of Information Technology, Chengdu, China

**Keywords:** Electrical and electronic engineering, Computer science

## Abstract

In recent years, unmanned aerial vehicles (UAVs) have undergoing experienced remarkable advancements. Nevertheless, the growing utilization of UAVs brings forth potential security threats to the public, particularly in private and sensitive locales. To address these emerging hazards, we introduce a low-cost, three-stage UAV detection framework for monitoring invading UAVs in vulnerable zones. This framework devised through an exhaustive investigation of the Chinese UAV market. Various scenarios were examined to evaluate the effectiveness of the framework, and it was subsequently implemented on a portable board. Experiments demonstrated that the proposed framework can detect invading UAVs at an early stage, even in *stealthy* mode. As such, the framework has the potential to be applied in the formulation of a portable surveillance system for a UAV-restricted region.

## Introduction

In recent times, there has been a decrease in the technical threshold and manufacturing cost of Unmanned Aerial Vehicles (UAVs), which has made it possible for the general public to use them for various purposes, such as toy play, photography, risk monitoring, scientific research, and the like. However, the use of UAVs has also exposed us to potential threats in areas like personal privacy, national security, and airspace management^1^. For instance, a drone crashed at the White House, while another interfered with a U.S. Open tennis tournament. On March 29, 2016, a drone collided with a Lufthansa plane near the Los Angeles International Airport (LAX), raising concerns about the safety of government buildings, air traffic, and other facilities^2^. Similarly, in China, an incident of drone interference with airport flights at Chengdu Shuangliu Airport in 2017 brought the issue of public safety of drones to the forefront, prompting authorities to focus on regulating drone flights^3^.

In order to mitigate these threats, the Chinese government has proposed a number of effective regulations aimed at managing UAV-related issues. For instance, the Civil Aviation Administration of China (CAAC) has stipulated that drone operators must possess a formal flight license, while fly-restricted zones have been established around flight routes, airports, and military areas. Moreover, drone monitoring stations have been set up during major events.

Nonetheless, the implementation of these regulations is no easy feat. In fact, a significant number of drones remain unregistered, and many low-cost drones can still fly unimpeded in fly-restricted areas, as they are difficult to detect and control. Consequently, there is an urgent need for the Chinese government and relevant departments to devise methods for detecting unauthorized UAVs and obtaining real-time information on them, including their identification, manufacturer, and remote connection. A desired UAV detection framework should be capable of identifying intruding UAVs in restricted airspace at an early stage, allowing for the prompt location and neutralization of the intruding drone, as well as identification of its owner. Additionally, it is of great importance to obtain information from intruding drones for the purposes of forensics. Useful information such as the drone’s identification, manufacturer, and remote connection can be leveraged in UAV location and UAV owner tracking, and can also serve as evidence in legal proceedings.

Due to the increasing awareness of the potential hazards posed by unauthorized Unmanned Aerial Vehicles (UAVs), UAV detection technology has become a focal point for countries. Current research on drone monitoring has evolved in various directions. The primary concept behind drone detection is to identify specific characteristics of the drones that distinguish them from other objects. In the literature, detection schemes that refer to different characteristics of UAVs have been proposed, of which the most popular solutions are broadly classified into vision-based, sound-based, radar-based, radio-frequency-based (RF-based), and WiFi-based technologies^4^.

A vision-based UAV detection system employs cameras to capture images containing UAVs and applies object recognition algorithms^5,6^ to identify UAVs from the video frames. The success of vision-based detection highly depends on the spatial resolution of commercial cameras. A drone far away from the camera will contribute only a few pixels in the video frame and is difficult to distinguish from other objects in the air, such as birds and insects. Furthermore, vision-based UAV detection is only possible when the target is in the line of sight and lighting conditions are good.

A sound-based UAV detection system^7,8^ employs microphones to capture acoustic information from motors equipped in nearby UAVs and extracts audio data features in either the time domain or frequency domain. Machine learning algorithms are also applied for acoustic feature recognition for UAVs^9^. Sound-based UAV detection is sensitive to environmental noise; therefore, it is not suitable for complex urban areas.

A radar-based UAV detection system^10,11^ utilizes characteristic features and spectral correlation functions from electromagnetic echoes of a Doppler radar and applies deep belief networks (DBN) to identify UAVs from other objects^12^. A major disadvantage of radar-based detection is that the radar signals could be blocked by buildings and other objects; therefore, it may not be suitable in urban areas. Moreover, the radar sensor is typically expensive and power-consuming.

Opposite to the three above-mentioned UAV detection methods, which are not effective in crowded urban areas, both RF-based and WiFi-based UAV detection systems^13–18^ are designed to detect the radio signal between UAVs and their control devices.Since UAVs communicate to their control device on a particular wireless radio channel frequently for the purpose of UAV piloting and video streaming^19^, it is possible to detect UAVs according to the features of radio signals.The most common frequency bands used by UAVs are two industrial, scientific and medical (ISM) radio bands located around 2.4 GHz and 5.8 GHz, which can be used without a license. When contrasted with the aforementioned detection methodologies, the RF-based approach proves more resource-effective, solely necessitating a radio frequency receiver, such as the Universal Software Radio Peripheral(USRP), thus offering a favorable power consumption profile, particularly in relation to radar systems.

In the RF-based approach, researchers^13,14^ have used raw RF data for research and proposed innovative methods for detecting and identifying drones based on RF signals transmitted from drones to controllers. They have developed deep neural network architectures to collect, analyze, and record the raw RF signals of different drones in various flight modes. These methods involve comparing power spectral density (PSD) with a PSD model trained as a regression task and using this model to detect and identify intruding drones, including detecting their presence, appearance, and type.Similarly, other researchers have studied the detection and classification of RF signals from different UAV controllers, even in the presence of wireless interference from Wi-Fi and Bluetooth sources. They have utilized radio frequency fingerprints and machine learning-based classification techniques to identify drones. Another research work proposed a new machine learning approach that utilizes RF data and network packet measurement to identify the unique characteristics of Wi-Fi devices, enabling the distinction between Wi-Fi drones and standard Wi-Fi devices in urban environments^17^. This method uses joint data measurement to identify the presence of drones in denied Wi-Fi environments. In contrast to these approaches, which often involve complex calculations and modifications of raw RF data, Bisio et al.’s^15^ method leverages network data traffic packets alone, collected using wireless sniffing tools, to detect the presence of intruding drones in the surrounding environment. In a WiFi-based UAV detection system, surveillance is carried out by monitoring the WiFi traffic exchanged between UAVs and their control devices. This method holds appeal due to the utilization of low-cost and power-efficient WiFi sensors, as well as its inherent robustness against environmental interference and noise.

**Figure 1:**
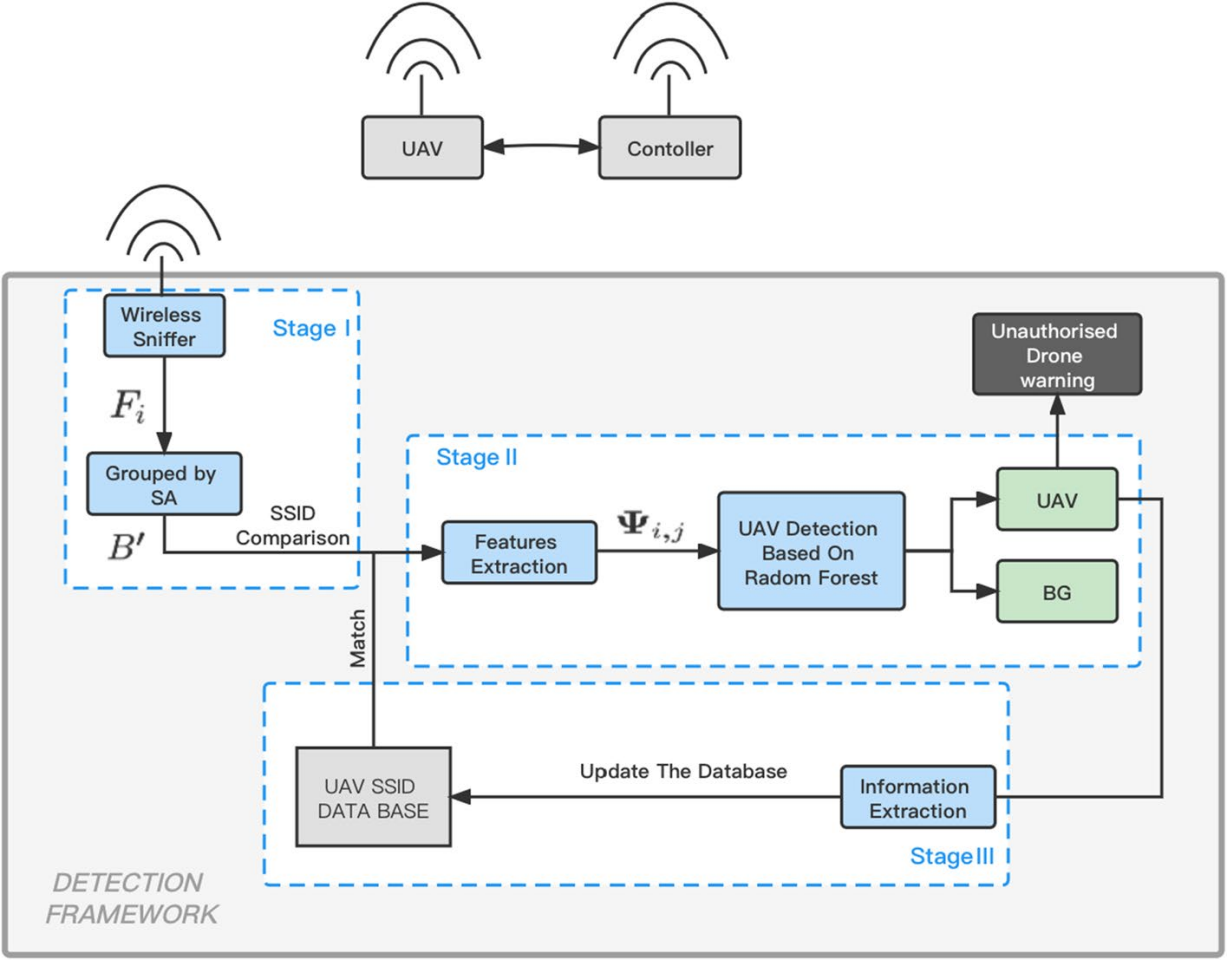
Low-cost UAV detection framework.

In China, the utilization of drones continues to expand, creating a significant business opportunity for the drone industry^20^. An increasing number of universities and research centers are undertaking research and development in consumer-grade drone technology. Moreover, the growing popularity of short-form video publishing platforms such as TikTok has resulted in a surge in both the import and export markets for aerial drones. According to BI Intelligence, the global UAV market is projected to grow at a compound annual growth rate (CAGR) of 19% between 2015 and 2020, with a 5% increase in the military sector^2^. As stated in^20^, China exported 891,000 UAVs in 2015, a 427.2% increase from the previous year, and imported 14.5 million UAVs, a 1350% increase from 2014.

Considering the characteristics of the Chinese UAV market,we have concluded that the WiFi-based method is a suitable approach for detecting unmanned aerial vehicles (UAVs) in China. However, the state-of-the-art WiFi-based UAV detection poses unique challenges, including: (1) The computational complexity of the UAV detection method must be low to facilitate the development of portable UAV detection devices. (2) Real-time UAV detection during the early stages of detection is critical. Current machine-learning detection methods^21^ optimize their model by minimizing detection accuracy while increasing the detection time, resulting in long detection times that are unacceptable for real-time detection. For example^22^, conducted experiments on the length of different time windows and found that statistical time was most effective at 5 s. (3) It is necessary to detect non-video-streaming UAVs. Some invading UAVs attempt to avoid detection by disrupting real-time video streaming, making them difficult to detect. (4) There is a lack of a database containing information on invading drones. The government should establish an information database on invading drones to enable the tracking, control, and forensic investigation of invading UAVs and their owners.

The present manuscript unveils a groundbreaking methodology for identifying the majority of unmanned aerial vehicles (UAVs) in China. Our innovative approach harnesses the inherent attributes of video streaming to swiftly detect UAVs and their associated devices within the range of WiFi sniffers. We have skillfully accounted for the distinctive characteristics exhibited by WiFi traffic emanating from UAVs and seamlessly integrated machine learning algorithms to identify these devices within the surveillance area. Upon detecting a UAV, we conduct a meticulous comparison against a dedicated UAV database, meticulously documenting crucial details such as the Mac address, manufacturer name, model type, and signal power of unlicensed UAVs. Diverging from conventional radio frequency-based methods for UAV detection, our framework is purposefully designed to embody cost-efficient hardware and computing solutions. Consequently, we capture traffic packet data via wireless network cards, circumventing the need for intermediate reception of raw radio frequency data. The intricacies of demodulation and decoding, which typically rely on pricier radio frequency and intermediate frequency hardware, no longer constitute a factor. In our thorough complexity analysis experiment, we scrutinized the memory usage and computational intricacies of the UAV detection framework. The results unequivocally demonstrate the remarkably low complexity of our approach. This framework significantly simplifies integration into lower-cost hardware devices and portable handheld devices.

Our main contributions are summarized as follows:

- We have proposed a novel learning-based framework for low-cost detection of invading unmanned aerial vehicles (UAVs). The framework comprises three stages: WiFi data sniffer and pre-processing, UAV classifier, and UAV database maintenance. Our framework is designed to run on a low-cost portable device and is capable of detecting invading UAVs at a very early stage.
- WiFi traffic flow traces are captured and separated into different groups based on their source MAC address within a short time slot. This modification enables us to shift the problem from ‘detecting UAVs within the surveillance area’ to ‘classifying whether a captured traffic trace belongs to a UAV’. This approach helps to reduce the number of features, making the computational complexity of the proposed method low. Several discriminative statistical features are then extracted from public headers in each group so that the algorithm can be applied even if the UAV communication is encrypted.
- We have established a UAV dataset that includes both classic mode (video-streaming) and *stealthy* mode (non-video-streaming) so that our framework is capable of detecting both normal UAVs and *stealthy* UAVs that disable video streaming.
- Drawing inspiration from Bisio et al.^15^, we have devised and incorporated our framework and algorithm into a compact device featuring an integrated CPU and WiFi chipset. We gathered authentic encrypted WiFi data traffic from a plethora of non-drones as well as six consumer drones, and conducted thorough performance evaluations of our innovative approach and Bisio et al.’s algorithm^15^ across a diverse range of illustrative scenarios.

The remainder of this paper is structured as follows: The low-cost UAV detection framework is proposed in detail in “Low-cost UAV detection framework”. “Data collection and predictor training” describes the collection and analysis of real-world datasets and the training of the model, while “Performance evaluation” conducts extensive experiments and performance evaluations. “Experimental implementation” describes the application of our proposed algorithm in concrete terms, and “Conclusion” concludes the paper.

## Low-cost UAV detection framework

The proposed three-stage invading UAV detection framework is briefly shown in Fig. 1. In the pre-processing stage, WiFi packets are filtered and grouped based on their source and destination addresses. In the feature extraction stage, four simple features are extracted from each packet group, and a random forest classifier is used to identify if the group belongs to an invading UAV. Finally, the identified UAVs are documented in a database indexed by their source addresses, and more characteristics such as model type, serial number, and manufacturer name are extracted to aid in identifying and conducting forensics on the UAV users. To sum up, the framework can help organizations and authorities to detect and identify unauthorized WiFi based UAVs and take necessary actions to ensure security and safety.

### Stage I: WIFI data sniffer and prepossessing

**Figure 2:**
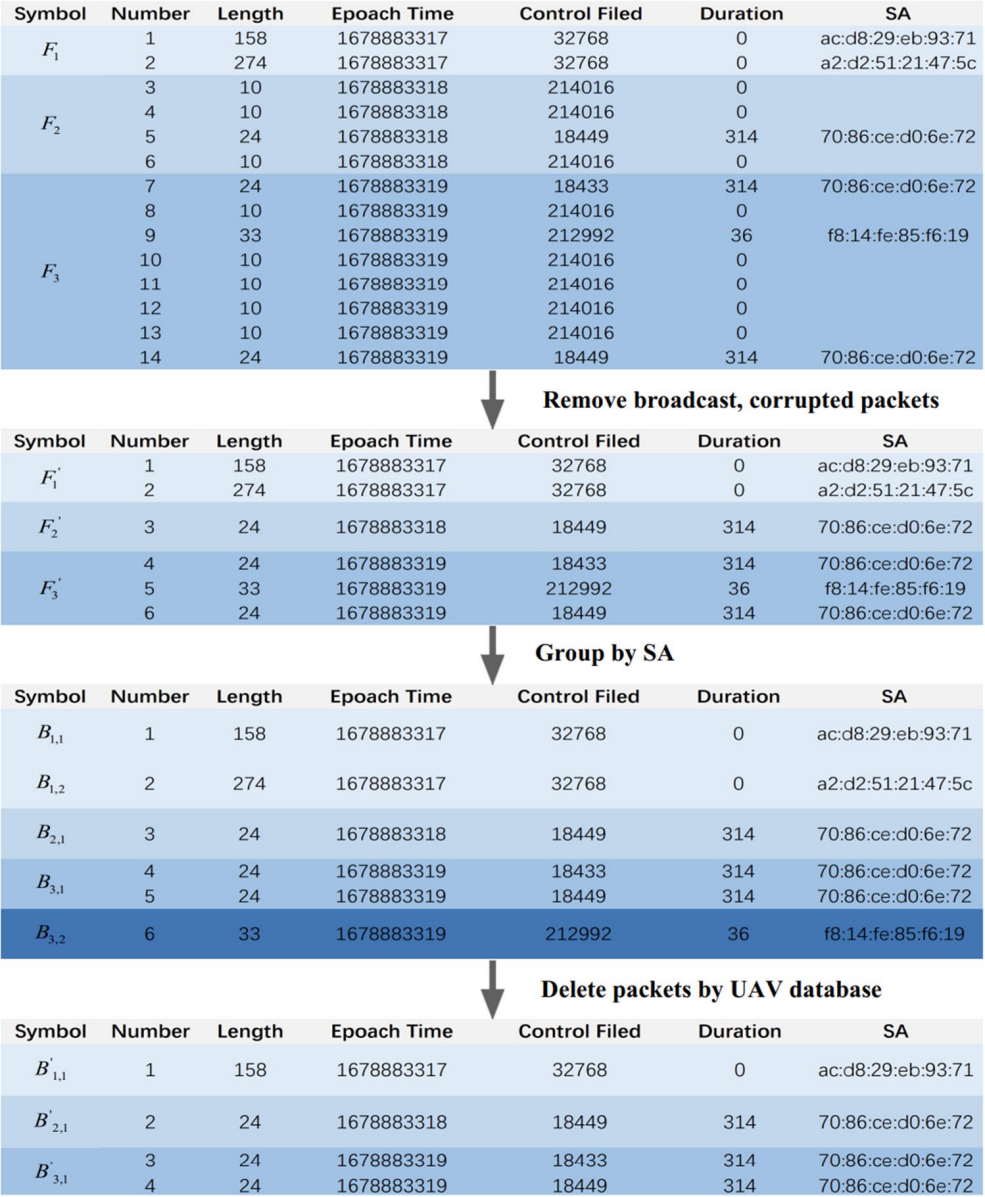
WIFI data pre-processing.

In the initial phase of our UAV detection framework, we employ a novel approach that involves the continuous capture of WiFi network traffic across all channels, partitioning it into 1-s time slots. This key innovation is designed to prioritize rapid response for the early-stage detection of invading UAVs. While it’s recognized in^15^ that employing a longer time window can enhance detection accuracy and stability, our framework’s unique emphasis on quick response sets it apart. This differentiation is instrumental in our ability to achieve a significantly higher level of detection accuracy, even in scenarios with minimal time for response.

To streamline the computational complexity, we have introduced an innovative data reduction strategy. We remove all broadcast packets (e.g., 802.11 beacon frames), corrupted packets, and packets with only the receive address (e.g., 802.11 ACK frames) from the captured WIFI traffic flow trace $F_i$, resulting in a cropped flow trace $F'_i$. On average, $F'_i$ is only 44% of the size of $F_i$, and it can be even smaller if there are several WiFi nodes nearby. This compact representation ensures the efficient analysis of data, particularly in situations involving multiple proximate WiFi nodes.

Encrypted or non-encrypted packets can be transmitted from an invading UAV. Although encrypted packets provide limited information, we can still obtain their public header, including the source Medium Access Control (MAC) address (SA), destination MAC address (DA), transmitter MAC address (TA), receiver MAC address (RA), basic service set identifier (BSSID), packet length, epoch time, and other MAC header information, such as frame type, (control, management, or data), sequence number, and duration/connection ID.

Furthermore, we introduce an inventive grouping and filtering mechanism within the cropped flow trace. We further divide the cropped flow trace $F'_i$ into several groups based on the SA of each packet. Let *B* *i*, *j* be the group belonging to the *j*-th SA in the *i*-th cropped flow trace. Before entering the next stage, we filter group *B* by comparing the SA of each group $B_{i,j}$ to our UAV database. We remove groups whose SAs exist in the database, resulting in $B'$, which contains groups with unknown SAs only.

For a more comprehensive elaboration on the WiFi data sniffer and pre-processing process, we will furnish you with a detailed guide containing step-by-step instructions on how to process the pre-acquired data depicted in Fig.  2. The control frame type is converted to decimal format for better representation. Presuming that the attribute bearing the source address a2:d2:51:21:47:5c/f8:14:fe:85:f6:19 is listed in the database and categorized as a drone.

### Stage II: UAV classifier

In this stage, four simple features are extracted from each group in $B'$, and machine learning based classifier is applied to decide whether the traffic group belongs to a UAV or not. Given the *i*-th ($m\in [1,M]$ where M is the total number of packets in this group) packet in a group $B'_{i,j}$, we can extract its packet length $p_{i,j,m}$, frame control field $f_{i,j,m}$ and duration $d_{i,j,m}$ from its public header. Then, for a particular group $B'_{i,j}$, four features called total number of packets $TN_{i,j}$, average packet length $AL_{i,j}$, root mean square value of frame control field $RF_{i,j}$ and root mean square value of duration $RD_{i,j}$, could be calculated sequently as

1 $$\begin{aligned}{} & {} TN_{i,j}=M \end{aligned}$$

2 $$\begin{aligned}{} & {} AL_{i,j}=\frac{1}{M}\sum _{m=1}^M p_{i,j,m} \end{aligned}$$

3 $$\begin{aligned}{} & {} RF_{i,j}=\sqrt{\frac{1}{M}\sum _{m=1}^M |{f_{i,j,m}}|^2} \end{aligned}$$

4 $$\begin{aligned}{} & {} RD_{i,j}=\sqrt{\frac{1}{M}\sum _{m=1}^M |{d_{i,j,m}}|^2} \end{aligned}$$

Finally, a feature vector $\Psi _{i,j}$ describing the *j*-th SA in the *i*-th cropped flow trace is defined by concentrating the four features together,

5 $$\begin{aligned} \Psi _{i,j}=\lbrace {TN_{i,j},AL_{i,j},RF_{i,j},RD_{i,j}}\rbrace \end{aligned}$$

and the feature vectors are inputted to a trained random forest classifier to identify whether this SA belongs to an invading UAV.

### Stage III: Invading UAV database maintenance

When an SA is identified as an unauthorized UAV, it is documented in a database for invading UAVs. The SA serves as the index of the database, and multiple characteristics are extracted from the WiFi traffic.

The invading UAVs in the database are classified by their SA. For each UAV, its model type and serial number can be retrieved from the Extended Service Set Identifier (ESSID) of the WiFi packets, and the manufacturer name can be determined for common UAV manufacturers. Additional details, such as UAV manufacturer, type, serial number, and DA, aid in identifying and conducting forensics on the UAV users. Supplementary information, such as signal power and frequency, can be utilized to further locate and track the invading UAVs.

## Data collection and predictor training

### Dataset preparation

In order to generate a discriminative random forest classifier, a dataset containing WIFI traffic of both UAV and non-UAV devices should be established and labeled correctly.

We obtained WiFi traffic data from six consumer unmanned aerial vehicles (UAVs) purchased from different manufacturers. A Lenovo R9000P laptop equipped with a wireless network interface card (NIC) was utilized to operate in promiscuous mode, monitor and capture WIFI network traffic. The monitoring NIC’s channel frequency was configured to match the operating channel of the UAVs, and Wireshark version 2.6.10 was utilized to capture the WIFI traffic data.

For each UAV, we captured WiFi packets for a total of 60–30 min while the UAV was streaming video, and an additional 30 min with the video transmitting feature disabled (to solely capture *stealthy* packets for the UAV). The raw captured data were then partitioned into 1-s intervals, further filtered and divided into several groups based on their source addresses, as demonstrated in “Stage I: WIFI data sniffer and prepossessing”. The resulting traffic flow traces were labeled “UAV”, and all flow traces were ultimately combined into a UAV dataset. Based on the dataset statistics, the initial count of raw captured data reached an impressive 1,618,063, which was significantly reduced to 8667 traffic flow traces after undergoing the pre-processing steps proposed by the framework. This pre-processing step also contributes significantly to computational efficiency.

Similarly, we captured non-UAV traffic flow using the same hardware to create a non-UAV dataset. We deployed two encrypted wireless routers that supported a variety of incoming and outgoing traffic types, such as web browsing, video streaming, online conferencing, and online gaming. The monitoring NIC was configured to operate on the same channel frequency and continued capturing data until the number of non-UAV traffic flow traces reached 9000, which was roughly equal to the number of UAV traffic traces.

Subsequently, the UAV and non-UAV datasets were merged, and 70% of the data was randomly selected for training the random forest predictor, while the remaining 30% was utilized as the testing dataset.

### Feature evaluation

**Figure 3:**
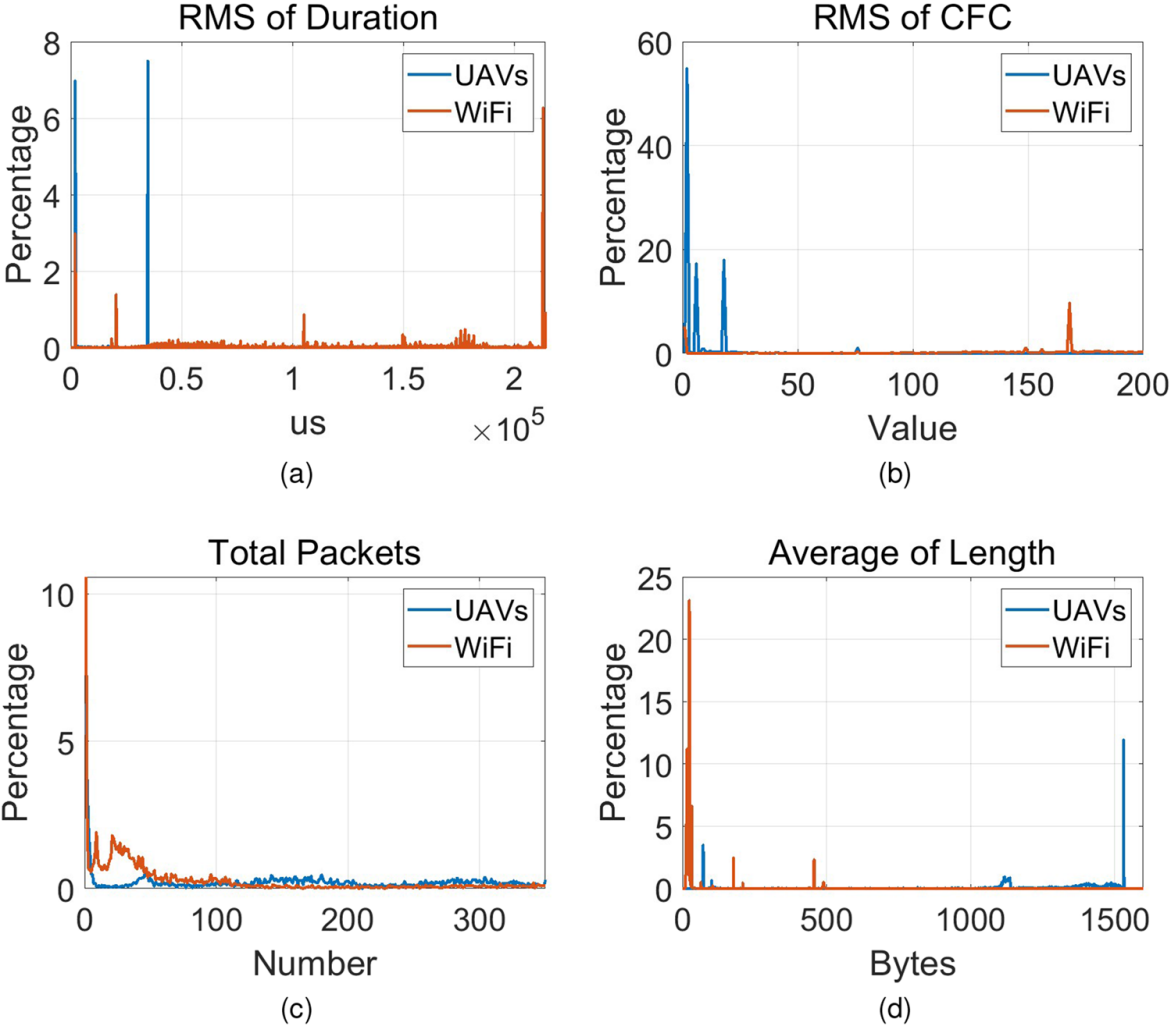
Distribution of the proposed features in the training dataset. (**a**) Root mean square value of duration. (**b**) Root mean square value of frame control field. (**c**) Total number of packets. (**d**) Average packet length.

The four features described in ““Low-cost UAV detection framework”” were then evaluated in the training dataset. Distributions of the four features are shown in the Fig.  3. According to Fig.  3, the proposed four features have certain discriminative capabilities for UAV and non-UAV traffic flows, hence they are expected to be appropriate input to machine learning-based classifier predictor for classification task.

### Predictor training

In this study, we utilized three commonly used classification algorithms, namely Random Forest (RF), K-Nearest Neighbors (KNN), and Support Vector Machine (SVM), for our experiments in UAV detection. These algorithms were chosen due to their well-established reputation in the field.

The Random Forest algorithm’s performance is influenced by the number of trees, which is a crucial hyperparameter. To optimize the performance while maintaining computational efficiency, we experimented with different numbers of trees. From our experiments, we found that a number of trees equal to 9 achieved excellent performance, with an accuracy rate of 99.91% and AUC of 1.00.

The SVM algorithm’s performance heavily depends on the selection of the kernel function, another essential hyperparameter. We explored linear, polynomial, and Gaussian (rbf) kernels, and after careful evaluation, we determined that the Gaussian kernel yielded the best results. The SVM model achieved an accuracy rate of 99.88% and an impressive AUC of 0.9998.

The KNN algorithm operates on the principle of proximity-based classification, assigning class labels based on the majority class among the K nearest neighbors in the feature space. The choice of K, denoting the number of neighbors to consider, greatly influences the algorithm’s performance. Through experimentation, we identified K = 2 as the optimal value, resulting in an accuracy rate of 99.86% and an AUC of 0.997.

To comprehensively analyze the performance of different classifiers, we conducted experiments with various parameters using the same dataset. We compared the accuracy and AUC values for KNN, SVM, RF, XGBoost^23^, Shallow Neural Network (SNN), and Convolutional Neural Network (CNN) models, as presented in Table 1. Notably, all models achieved accuracy rates over 90%, indicating their effectiveness in UAV recognition.

Among the models evaluated, RF, XGBoost, SNN, and SVM achieved near-perfect accuracy rates of approximately 99.9% or higher, with corresponding AUC values of 1.00. KNN also demonstrated strong performance, with an accuracy rate of 99.86% and an AUC of 0.997. However, the CNN model exhibited relatively lower accuracy (95.43%) and AUC (0.9978). This discrepancy may be attributed to the complexity and unique characteristics of the dataset, which presented challenges for CNN’s feature extraction.

Based on the overall performance, we selected the Random Forest algorithm as our framework for UAV recognition. Random Forests are widely recognized classifiers, consisting of multiple decision trees and utilizing an integrated learning approach called Bagging. In our implementation, the Random Forest model was trained using the TreeBagger function in MATLAB. We determined the optimal number of trees to be 9 based on the analysis of the out-of-bag classification error. Further increasing the number of trees did not significantly reduce the error rate. This parameter was consistently used throughout our experiments.

Additionally, we replicated Bisio’s algorithm^15^ using the Random Forest model as the benchmark. A detailed comparison of the performance between the two algorithms will be provided in the subsequent sections of the paper.

**Table 1:** Performance comparison of different models.

Algorithm	Accuracy (%)	AUC	Inference time(us)
KNN	99.86	0.997	4.9027
SVM	99.88	0.9998	0.5874
RF	99.91	1.00	23.1408
XGBoost	99.80	1.00	18.0269
SNN	99.74	1.00	6.5287
CNN	95.43	0.9978	7.7629

## Performance evaluation

This section presents a comprehensive performance analysis of our proposed drone detection framework. We replicated Bisio’s UAV detection method^15^ and quantified the advantages of our framework in terms of UAV detection speed and accuracy. Moreover, we conducted a thorough analysis of the complexity of the employed models and compared their inference performance. The following section outlines the specific experimental process.

### Experimental assessment of detection performance

We assessed the effectiveness of our proposed UAV detection framework and Bisio’s method^15^ by employing them to identify UAVs amidst heavy WiFi traffic flows across various detailed scenarios. The experiments were conducted using the MATLAB2023a platform and the Lenovo R9000P model operating on the WIN11 operating system. The experimental data of the four verification scenarios were collected using a MediaTek RT3070 NIC, with the collection device positioned 10 m away from the drone. To simulate a complex experimental environment, a WiFi interference source was placed 5 m away from the collection device. The four distinct experimental scenarios are described as follows:

1. Scenario-1: Multiple UAVs with Wifi background traffic.We positioned six flying drones, each controlled by an Android smartphone with video streaming enabled, above a clear yard on campus. Multiple WiFi routers in the vicinity generated normal background traffic. Our NIC operated in promiscuous mode, with the channel frequency set to match that of the UAVs’ operating channel.
2. Scenario-2: Multiple *stealthy* UAVs with background traffic. The experimental setup for this scenario was similar to Scenario-1. However, all UAVs disabled their video transmission and worked in *stealthy* mode to eliminate most of their traffic flows, deceiving the UAV surveillance system.
3. Scenario-3: Multiple *stealthy* UAVs with heavy background traffic. In this scenario, we disabled video streaming for all six UAVs. Additionally, we started an encrypted wireless router nearby with heavy incoming and outgoing traffic, such as video streaming, online video conferences, and FTP downloads.
4. Scenario-4: NIC active scan mode. This scenario simulated the common usage of our framework, in which the NIC scans all 24 channels in the 2.4 GHz and 5 GHz spectrums cyclically and captures packets from all channels. Six UAVs were set to *stealthy* mode and worked on different channels.

During the validation phase, several popular statistical metrics are utilized to objectively evaluate the performance of the proposed model in the above scenarios. Probability of detection (POD), cumulative success index (CSI), and false rate (FAR) can be formulated as follows^24^:

6 $$\begin{aligned}{} & {} POD = \frac{n_{success}}{n_{total}} \end{aligned}$$

7 $$\begin{aligned}{} & {} CSI = \frac{n_{success}}{n_{total} + n_{false\,alarm}} \end{aligned}$$

8 $$\begin{aligned}{} & {} FAR = \frac{n_{false\,alarm}}{n_{success} + n_{false\,alarm}} \end{aligned}$$

where $n_{success}$,$n_{false}$,and $n_{total}$ are the number of *success*, ${false\,alarm}$, and *total* of UAV traffic flows. *Success* means a traffic flow trace is identified to UAV correctly while ${false\,alarm}$ means a background traffic flow trace is reported as UAV. Values of POD, CSI and FAR range in [0,1].

Besides the above metric, F-score is a common measure of a learning based classification algorithm. F-score is a statistical approach to determining accuracy, taking into account both precision and recall, where Precision is the number of volumes added correctly and Recall measures how many volumes are missed. They are defined as follows:

9 $$\begin{aligned}{} & {} Precision = \frac{TP}{TP + FP} \end{aligned}$$

10 $$\begin{aligned}{} & {} Recall = \frac{TP}{TP + FN} \end{aligned}$$

11 $$\begin{aligned} F-Score = \left( 1 + \beta ^{2} \right) \frac{Precision*Recall}{\left( \beta ^{2}*Precision \right) + Recall} \end{aligned}$$

where TP, FP and FN denote true positives, false positives and false negatives; $\beta$ is set to 1 so that F-score combines the results of precision and recall. A high F-Score indicates that the test model is effective.

Table 2 reports the evaluation results of the proposed model in the training dataset and four mentioned scenarios, and then shows the performance of the algorithm of Bisio et al.^15^ in the same experimental scenario. In the algorithm we propose, POD and CSI values are relatively high while FAR values are low in all scenarios, indicating most UAVs could be detected correctly with acceptable false alarm rate.

Within the four scenarios, Scenario-1 represents the optimal case where all invading UAVs enable video streaming. These packets possess distinct characteristics, resulting in a detection probability as high as 99.93%. This, in turn, affirms the proposed model’s anticipated resilience against various types of UAVs, even if they are not incorporated in the training dataset. In Scenario-2, fewer packets are transmitted and received in *stealthy* mode, which drives the algorithm to be less prone to packet loss within the specified time window, thereby resulting in 100% POD. Performance evaluations of Scenarios 3 and 4 illustrate that it is more challenging to detect a *stealthy* UAV amidst heavy background traffic. Nevertheless, the POD values for Scenarios 3 and 4 remain above 99%, demonstrating that our framework can detect most UAVs that operate in stealthy mode. However, the F-scores for Scenarios 3 and 4 decline to 0.9926 and 0.8992, respectively. In order to assess our model’s ability to detect *stealthy* UAVs, we have provided the confusion matrix of UAV identification in Scenario-3:

12 $$\begin{aligned} \begin{bmatrix} TP &{} FN \\ FP &{} TN \\ \end{bmatrix} = \begin{bmatrix} 400 &{} 3 \\ 5 &{} 895 \\ \end{bmatrix} \end{aligned}$$

It is evident that all *stealthy* UAVs are identified correctly, with only 5 background traffic traces being misclassified as UAVs and counted as false alarms. UAV identification in Scenario-3 represents an unbalanced class, owing to the fact that a *stealthy* UAV transmits packets only, and the total amount of UAV traffic traces is significantly lower than that of background traffic. Additionally, we employed an encrypted wireless router with heavy wireless traffic in Scenario-3, exacerbating the class imbalance. This explains why the false alarm rate (FAR) of Scenario-3 is slightly higher. Scenario-4 is the typical usage condition of our framework, wherein six *stealthy* UAVs within different wireless channels attempt to infiltrate the surveillance area. Our model attains a POD of 99.41% and an F-score of 0.8992. We have successfully implemented the famous algorithm^15^ as a benchmark while the same training set is applied to optimize the algorithm. As demonstrated in Table 2, Bisio’s^15^ performs slightly worse in all the scenarios. More precisely, FAR of Bisio’s^15^ is 0.1299 while FAR of our algorithm is 0.0123. It indicates that Bisio’s^15^ introduces more false alarms while it processes with *stealthy* UAVs and encrypted background traffic.

**Table 2:** Performance results of prior algorithm related to UAV detection in various scenarios.

Experimental scenario	POD		CSI		FAR		F-Score	
	Ours	Bisio’s	Ours	Bisio’s	Ours	Bisio’s	Ours	Bisio’s
Training	1	1	0.9999	0.9989	0.0001	0.0011	0.9999	0.9994
Scenario-1	0.9993	1	0.99993	0.9795	0	0.0205	0.9997	0.9897
Scenario-2	1	0.9931	1	0.9611	0	0.0325	1	0.9802
Scenario-3	0.9926	0.9911	0.9804	0.8634	0.0123	0.1299	0.9901	0.9267
Scenario-4	0.9941	0.9898	0.8169	0.7902	0.1792	0.2033	0.8992	0.8828

### Complexity analysis

The complexity of the proposed framework and Bisio’s^15^ is then discussed. Our framework consists of four features which are calculated from a 1-s time slot. Among the four features, feature (1) is a single assignment operation of complexity *O*(1), while the other three features need one mean calculation and two root mean square calculations with a complexity of *O*(*n*). In contrast, Bisio’s^15^ requires six assignment operations and four calculation operations (encompassing mean, root mean square, and summation) from a 5-s time slot. Consequently, complexity of feature calculations for both algorithms is *O*(*n*), where *n* denotes the number of input data. However, our method need a 1-s time slot capturing only, and our method can decrease the packets by up to 44%, as has been shown in “Low-cost UAV detection framework”. Therefore, it is expected that complexity of the proposed framework is roughly 1/10 of Bisio’s^15^.

Additionally, we conducted complexity analysis on the six models referenced in the previous section^25^. The experimental data used are from the subsection described earlier. For K-Nearest Neighbors (KNN) during inference, the algorithm computes the distance between the test instance and all training instances, with a complexity of *O*(*n*), where n is the number of training instances. Furthermore, KNN involves distance sorting, which has a time complexity of $O(n*log n)$. In the inference process of Support Vector Machine (SVM), the algorithm computes a decision function by evaluating the kernel function of the support vector. The complexity is dependent on the number of support vectors and the computational cost of the kernel function. For the nonlinear SVM with Gaussian radial basis function (RBF) kernel function employed here, the complexity is *O*(*k*), where k represents the number of support vectors. In the case of Random Forest (RF), inference involves aggregating the predictions of each decision tree. The complexity scales linearly with the number of trees (T) and depends on the tree depth. For a balanced RF, the complexity is approximately $O(T*logn)$. XGBoost utilizes an ensemble of decision trees, aggregating their predictions. The complexity scales linearly with the number of lifting rounds and the depth of the tree. Shallow neural networks typically exhibit a complexity of *O*(*d*), where d represents the number of features. The network performs a series of matrix multiplications and activation function evaluations. As for Convolutional Neural Networks (CNNs), their complexity scales linearly with the number of layers and the size of the input vector. The network applies convolution and pooling operations, followed by fully connected layers. The complexity is contingent upon the specific architecture and input vector size.

Using the experimental data described in the previous section, with the aforementioned platform, each model performed 1000 inferences and the results were averaged. This allowed us to obtain the actual time required for a single inference of each model, as shown in Table 1. According to the experimental results, the SVM model demonstrated the shortest inference time. It is worth noting that the complexities mentioned here offer a general understanding of the computational requirements of each algorithm. The actual inference time is provided in Table 1, where we performed 700,000 inferences for each model to obtain the average inference time. From the experimental results, we observed that the SVM model efficiently detects the presence or absence of drones, with an inference time of less than 1us. Similarly, the single inference time of KNN, SNN, and CNN also falls within the 10us range. Only Random Forest (RF) and XGBoost exhibit slightly slower inference times. The inference complexity of these two models is influenced by the depth of the trees. However, in terms of accuracy, the Random Forest algorithm achieves the highest performance.

## Experimental implementation

In scenario 4, through experiments we found that an average of 29 traditional traffic packets are captured from the network card per second, and the average length of a single packet is 51 bytes, which is about 1488 bytes. Through the preprocessing and packet filtering in the proposed framework, the average length of each packet is 51 bytes. 16 valid traffic data packets, about 828 bytes, are obtained in seconds. Feature extraction is performed on the valid traffic data group to obtain a feature vector to detect the presence of a drone. Taking the random forest model as an example, the model size is about 148,480 bytes. Ignoring the size of the feature vector, our proposed framework requires about 150,796 bytes of memory for a single run. This will allow us to deploy our framework in embedded boards with lower computational costs. Our framework is then implemented in a portable board. Specifically, the board consists of a quad-core ARM Cortex-A55 CPU, 4GB memory, and 128GB eMMC storage. A MediaTek RT3070 NIC is inserted into the board as monitoring device. An version 20.04 ubuntu embedded system is running in the board, and Librf^26^, a C++ implementation library, is introduced to implement the *randomforest* predictor.

**Figure 4:**
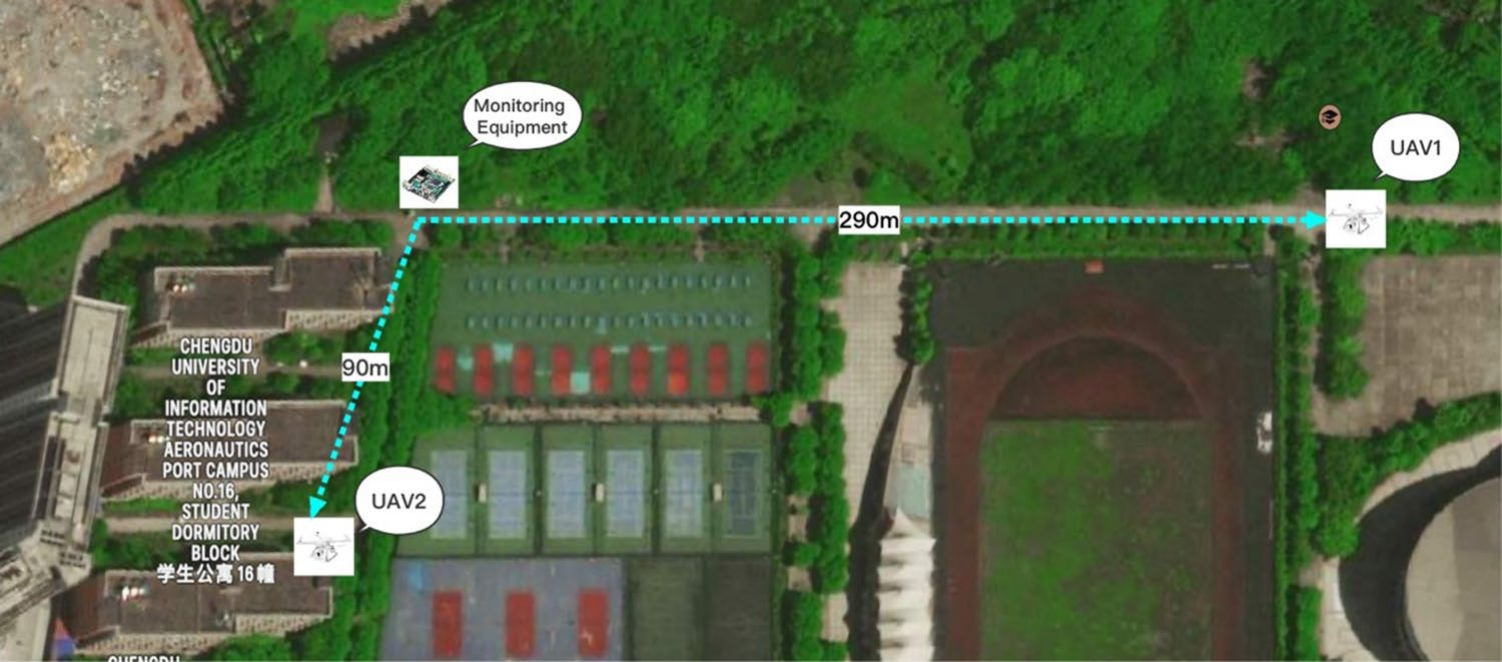
Range test of the proposed UAV surveillance system.

We conducted an experiment on our campus to evaluate the maximum detection range and detection time of the proposed framework. To achieve this, we directed the antenna towards two UAVs, one in line-of-sight and the other obstructed by a five-story building and some trees. The outcome depicted in Fig. 4 shows that the detection range for the two UAVs was 280 m and 90 m, respectively.

Table 3 displays the response time to identify invading UAVs, with $t_{d}$ representing the time taken to capture UAV packets into 1s time slot, and $t_{p}$ and $t_{f}$ indicating the duration of data pre-processing and *randomforest* predictor, respectively. The results confirm that our framework is computationally efficient, with detection time primarily dependent on the duration of capturing Wifi traffic. Since we set the length of capturing time slot to 1 s, we can always anticipate the identification of an invading UAV within 1 s. Moreover, our framework is capable of identifying a UAV even if only 3~4 UAV packets are present within the 1s time slot (as most of the packets are lost due to the long transmission range).

**Table 3:** Detection performance of far-away UAVs.

	Number of UAV packets	$t_{d}$	$t_{p}$	$t_{f}$	$t_{total}$
Line-of-sight UAV	4	16.45 ms	0.203 ms	1.775 ms	18.428 ms
Non-line-of-sight UAV	3	367.68 ms	0.283 ms	0.259 ms	368.222 ms

**Figure 5:**
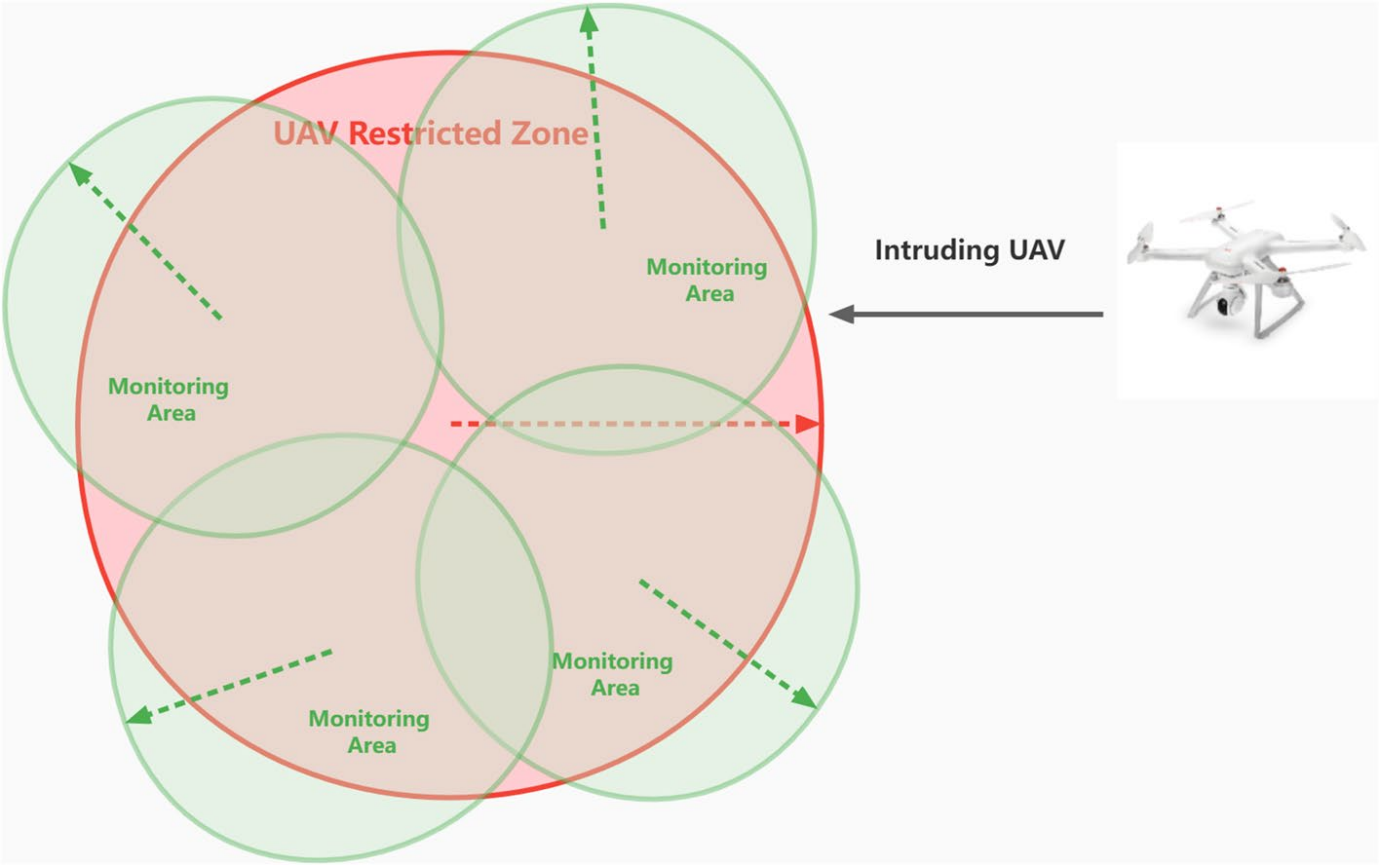
Distributed surveillance system consisted of multiple surveillance devices.

To expand the detection range of UAVs to meet the requirements of airport surveillance, we are contemplating a distributed surveillance system. This system comprises numerous portable detection boards and a centralized data center, as illustrated in Fig. 5. Whenever an integrated board identifies an encroaching UAV, the data will be transmitted to the central data center via cellular data network, where an invading UAV database is maintained and warnings are generated.

## Conclusion

This research delves into the analysis of the UAV market in China and proposes a cost-effective UAV detection framework for monitoring the incursion of UAVs in vulnerable areas, such as airports.

The suggested learning-based UAV detection framework acquires four distinct statistical features from public headers of WiFi traffic trace. A UAV dataset, which includes video streaming traffic and remote control signals, is developed and employed to train a random forest predictor offline. As a result, the predictor is anticipated to identify encroaching UAVs at an early stage. Additionally, the previous algorithm is duplicated, and the prediction accuracy and time complexity of the two algorithms are juxtaposed in the same environment for comparison. The outcomes indicate that our proposed framework is more advantageous in practical application scenarios.

The framework is assessed in various scenarios. In the case of UAVs that permit video streaming, POD reaches a staggering 99.93%, and the F-score is 0.9997. Conversely, when *stealthy* UAVs are detected in unknown channels, and video transmission is disabled, POD decreases to 99.41%, while the F-score is 0.8992. Based on the evaluation results, the proposed framework is demonstrated to effectively detect encroaching UAVs with satisfactory accuracy.

The framework does not necessitate any prior knowledge of unmanned aerial vehicles (UAVs). In contrast to DJI and other drone manufacturers who independently devise video transmission protocols, our proposed framework is specifically designed for drones that are equipped with a universal WiFi chip and utilize the 802.11 protocol for video streaming. Thus, this framework is not limited to detect UAVs that listed in the training dataset. In the experimental scenarios, we utilize drones that go beyond the scope of the training dataset, and our test scenarios encompass drones that were not included in the training set. This allows us to vividly demonstrate the universality of our framework.

The framework is realized on a portable board, which integrates a WiFi chip with an omnidirectional antenna to establish a UAV surveillance board. An experiment is conducted on campus, and the results exhibit that the board can detect encroaching UAVs within 300 m and 90 m in the line-of-sight and non-line-of-sight conditions, respectively. The surveillance system’s response time for line-of-sight and non-line-of-sight conditions is 18.428 ms and 368.222 ms, respectively.

The surveillance board is anticipated to be extended to a distributed surveillance system, which comprises multiple portable surveillance boards and a data center so that the detection range could cover the entire UAV restriction region of the airport in the future. Furthermore, we hope this study will inspire researchers to explore unauthorized UAV detection of varying transmission types.

## Data Availability

The datasets generated during and/or analysed during the current study are available in the IEEE Dataport repository.(https://dx.doi.org/10.21227/enfv-kx52).
